# Beyond Methodology: The Role of Reflexivity in Ethical Research That Benefits Marginalised Participants and Communities

**DOI:** 10.1177/15562646261463356

**Published:** 2026-07-09

**Authors:** Bethlehem Tekola

**Affiliations:** 1 Research Fellow in Qualitative Research, Health Services & Population Research, Institute of Psychiatry, Psychology & Neuroscience, 4616 King's College London, London, UK

**Keywords:** Reflexivity, research ethics, marginalised communities, qualitative research

## Abstract

Reflexivity is often framed in qualitative research methods literature as a means of enhancing methodological rigour and research quality. However, less attention has been paid to whether research that meets such standards is also ethically responsive to the needs of participants and communities. In this paper, I critically examine the role of reflexivity in advancing ethical research practices, particularly in studies involving marginalised groups and communities. Drawing on Indigenous and decolonial scholarship, together with insights from my own long-term engagement with marginalised groups and communities, I propose an approach to reflexivity that is community centred and ethically oriented. I further explore the limits of reflexivity in addressing power asymmetries and ensuring that research produces knowledge that matters to researched communities. This paper contributes to ongoing debates in qualitative inquiry by repositioning reflexivity not only as a marker of rigour but as a practice with ethical and political significance for research.

**What is already known**

1. Reflexivity is widely recognised in qualitative research methods literature as a means of enhancing methodological rigour and quality.
2. Reflexivity encompasses both methodological and ethical dimensions.
3. Ethical reflexivity extends beyond methodological quality by considering participant rights, relationships and broader social implications.

**What this paper adds**

1. Reframes reflexivity as a community centred practice that can actively benefit marginalised participants and communities.
2. Offers reflections on what ethical reflexivity that centres the researched community might look like.
3. Critically examines the limits of reflexivity in promoting research that is genuinely meaningful and accountable to participants and communities.

## Introduction

There are different conceptualisations of reflexivity across disciplines. But reflexivity is often defined as a way for researchers to critically reflect on their role in the research process and the knowledge they produce. A substantial body of qualitative research methods literature frames reflexivity as a marker of rigour and research quality. Alejandro and Stoffel (2024, p. 331) write, “Qualitative researchers have promoted reflexivity on the basis that it improves the quality of qualitative research.” However, it is not always clear whether “good” quality qualitative research also means ethically sound. Woods (2019, p. 462) observes, although reflexivity is widely regarded among qualitative researchers as essential for ensuring rigour and quality in research, “social scientists are also often vague about the place of ethics in this process.” Alejandro and Stoffel (2024, p. 342) suggest a multidimensional and inclusive approach to quality in qualitative research that includes, among other things, ethics. They argue that ethics should be central to what counts as “good” qualitative research, stating, “we believe that non-ethical research is better viewed as ‘low-quality’ by definition.”

This concern with ethics is not new. Earlier, Guillemin and Gillam (2004) explore the potential of reflexivity as a resource for ethical research. They distinguish between two kinds of ethics in research, which they called “procedural ethics” and “ethics in practice.” Procedural ethics refers to the rules and formal approvals researchers need to follow (such as university research ethics committee approval) before starting their research. Ethics in practice refers to the ethical issues that arise during the research process or the day-to-day ethical issues in the research process. Guillemin and Gillam argue that reflexivity links procedural ethics and ethics in practice by acting as a bridge between formal ethics rules (covering things like informed consent, confidentiality and avoiding harm) and the everyday decisions researchers make in the field. It does this by helping researchers think critically and act ethically when the rules alone are not enough or do not provide clear answers. Although, in the process of obtaining ethics approval, researchers need to show and plan ahead how they will protect participants in some situations, formal ethics approval processes cannot predict every situation that may arise in real life. This is where reflexivity becomes important. Reflexivity helps researchers to continually reflects on their actions, their relationship with participants and the effects of their choices and respond ethically in context.

Similarly, Von Unger (2021) discusses how researcher reflexivity not only serves analytical but also ethical purposes. For von Unger, ethical and methodological reflexivity are deeply connected but distinct in focus and purpose. Methodological reflexivity is concerned with the quality and validity of research itself. By contrast, ethical reflexivity expands the focus from methodological rigour to the social, political and relational consequences of research. She notes: “Ethical reflexivity is inclusive of methodological reflexivity but goes beyond it. It also takes the concerns and rights of participants into account, as well as the possible social and political implications of a study.” (2021, p. 199).

Within feminist and critical qualitative traditions, reflexivity is conceptualised as an ethical, epistemological and political practice (see, for example, Alcoff, 1991; Finlay, 2002; Haraway, 1988; Pillow, 2003). It involves researchers critically examining how knowledge is produced, represented and legitimised, and how power relations and positionality shape these processes.

In this paper, I build on these conceptualisations to explore the role of ethical reflexivity in conducting research that benefits marginalised participants and researched communities. Specifically, I ask the following two questions:

- How can researchers’ reflexivity benefit participants and communities?
- How can researchers practice reflexivity in ways that genuinely centre participants and communities?

In what follows, after outlining the influences that shape my thinking, I first offer reflections on what ethical reflexivity that centres the researched community might look like. I then explore the limits of reflexivity in supporting research practices that genuinely matter to participants and communities.

### Context

My thinking on reflexivity and ethics is informed by Indigenous and decolonial perspectives. These include Linda Tuhiwai Smith's (2021) perspective on research ethics as grounded in accountability (researchers are answerable to the communities being studied) and reciprocity (giving something back to the community), “a social justice orientation to research” (Brown & Strega, 2015, p. 4) which requires us to be ethically reflexive, “a community-focused approach” (Gaudry, 2015, p. 255), and “a framework of refusal within (and to) research” (Tuck & Yang, 2014, p. 225). In their 2014 book chapter, “R-words: Refusing research”, Eve Tuck and K. Wayne Yang discuss three axioms about social science research. One of the axioms, namely “Research may not be the intervention that is needed” is particularly relevant to my thinking in this paper and speaks directly to their “framework of refusal within (and to) research”. They challenge the assumption that research is inherently helpful:

“As social science researchers, we are trained to believe that research is useful (even if only vaguely useful) and that it can compel needed change (even if the theory of change is somewhat fuzzy, or flawed) … As such, when we see something that needs attention, resources, critique, or intercession, our initial inclination may be to conduct research on it. We generally do research to meet an unmet need. Yet there are far more instances than are commonly realized in which research is not the most useful or appropriate intervention.” (p.236)

Here, refusal is not simply a rejection of research but an ethical and political stance that questions when research itself may be extractive, inappropriate or a distraction from more necessary forms of action. Refusal, therefore, becomes part of an ethical orientation that ask whether research should be undertaken at all and who benefits from it. More broadly, these perspectives (“social justice-oriented research”, “a community-focused approach” and “a framework of refusal within (and to) research”) situate ethics and the research itself as inseparable from justice, accountability and relationality.

My reflections are also shaped by my multidisciplinary background and by many years of participating in studies involving marginalised groups and communities such as sex workers and children living in poverty. Having spent nearly a decade working on international health research projects (as a researcher from the Global South now based in the Global North), often in communities I relate to or am familiar with I have long been concerned with how research affects the communities being researched. In the research projects I have been involved in, participants have often asked whether research outcomes would lead to any meaningful changes in their lives or their communities. These experiences have led me to view reflexivity not only as a way of strengthening research credibility but also as a practice with ethical responsibilities. It also prompted me to ask whether reflexivity can be oriented more directly towards participants and communities and how researchers might work towards forms of reflexivity that prioritise benefit, accountability and justice.

## Towards a Reflexivity That Centres Researched Communities

I agree with Guillemin and Gillam (2004) and Von Unger (2021) that reflexivity should not be confined to knowledge production or valued solely for its ability to generate more credible and valid findings i.e., as a methodological tool. Reflexivity can and should be a key component of ethical research practice. I would also argue that reflexivity should not just be about researchers or the scientific community; it should also be about the researched. I always find it helpful to remember that just as researchers have hopes and expectations when we decide to carry out a study (such as publishing our findings in peer-reviewed journals and more broadly contributing to knowledge and informing practice and policy), research participants also have hopes and expectations when they agree to take part in a research project. In my experience, marginalised people and communities often participate in research with the hope that the research will bring about positive changes in their lives and/or their communities or in “the circumstances of those who are like them” Brown and Strega (2015, p. 7). In their article “Marginalized Communities and the Problem of Research Extraction”, Bothello and Bonfim (2023, p. 526) describe how, at the end of an interview in South Africa, a participant asked: “Wait – is that it? Are you coming back tomorrow?” Having just shared painful memories, the participant, they note, “revealed an implicit expectation that the interview would yield further interactions and reciprocity stemming from the exchange.”

Gani and Khan (2024, p. 4) draw on critiques of reflexivity in the literature to argue that reflexivity should be “less about oneself and instead focused on humility and practice-oriented disruption of unethical research”. That means reflexivity should not mainly focus on researchers talking about themselves (such as describing their identity and background). It should centre more on humility and on improving research practices in ways that prevent harm toward the researched community. It should also be judged by its practical value for the people taking part in the research not just by how well the researcher reflects on themselves. Researchers should, therefore, ask themselves: “What change does my reflexivity bring that actually helps or matters to the participants or the researched community?” or “How does my reflexivity make a difference for the people taking part in my research?”

### Ethical Reflexivity That Takes Participants’ Wishes Seriously

Research may not be the priority of people and communities. People and communities who have been researched again and again without seeing any positive change coming out of their participation in research projects may understandably reject research. Sultana (2007:, p. 381), for example, described an incident in rural Bangladesh where a woman dismissed her questions about participating in the research, saying, “Not another one of you people with more questions again”. Sultan explained that such reactions were especially common in villages that had been repeatedly studied by development agencies and NGOs. Refusals also occurred when Sultan clarified that she was not connected to projects providing tubewells or other water technologies, as many residents who urgently needed safe water had come to expect direct material benefits from researchers.

Ethically reflexive researchers must be prepared for communities to say, “We do not want research. We want other interventions.” Asking communities what they want requires being ready to accept that “research may not be the intervention that is needed” (Tuck & Yang, 2014, p. 236). For example, before writing funding applications, if researchers engage meaningfully with communities to ascertain whether new or further research is actually needed, researchers should then remain open to the possibility that these early engagements may lead to the abandonment of their research project. It is important to acknowledge that, although increasingly expected by funders, it may not be always possible to engage communities before writing funding applications for a number of reasons including lack of pre-award funding, insufficient time and challenges reaching communities (University of Glasgow, no date).

### Ethical Reflexivity That Requires Being Accountable and Responsible to the Researched Community

Ethically reflexive researchers should think carefully about and hold themselves accountable for the short- and long-term impact of their research on the researched community (Gaudry, 2015). This includes making sure that the outcome as well as the entire process of our research do not diminish in any way the wellbeing and health of research participants and their communities. It also means ensuring mutual benefits of participating in research (Chilisa, 2019; Kovach, 2021; Smith, 2021; Tobias et al., 2013; Wilson, 2008). That is, communities should receive something in return for contributing their knowledge, time and experiences. When researchers take knowledge or information from communities without meaningfully giving back, the research becomes extractive (Gaudry, 2011). Oyinloye (2021), who conducted ethnographic research with two rural Yorùbá communities in Northcentral Nigeria, provides a participant's account that illustrates frustration with outsiders who had previously visited the community. The participant noted that these outsiders asked detailed questions about their work and livestock but failed to deliver on promised improvements, leaving behind disappointment and frustration. Oyinloye (2021) further shows how departing a community without offering anything of value violates local ethical principles, stressing that any benefits should be both meaningful and determined by the community itself.

In collecting data, researchers may prompt people to speak up about their concerns, yet often fail to provide support, share the findings or follow up on the issues and needs identified in the research. As Sherwood and Anthony (2020, p. 43) put it, “Researchers open up wounds, deplete communities of their knowledge, and leave empty promises with no accountability.” In order to do non-extractive research, Kouritzin and Nakagawa (2018, p. 683) suggest that researchers must ask the following reflexive questions: “Whose questions are being asked?” “Am I the one who should be asking these question(s)?”

### Ethical Reflexivity That Requires Sometimes Stepping Back

Ethical reflexivity means recognising that we may not be the appropriate person to conduct a study even if we have the resources or the ability to do so (Hagen et al., 2023). We need to think carefully, as Brown and Strega (2015, p. 4) note, “how and where we are in relation to the contexts, communities and participants we study.” Such reflexivity should be able to make us say, “I am not the right person to do this research or to lead this research project, so I am not going to do it.” This reflexivity is not just about our competence as researchers but also about our relational positioning i.e., whether our relationship to a community, participants and a context enable or constrain ethical research. For example, a researcher might conclude that they are not the right person to conduct research if they lack the cultural, linguistic and historical knowledge needed to understand the community responsibly. A researcher might also conclude that they are not the right person to conduct research with certain participants (e.g., children) if they lack the training and skills needed to ensure the participants’ meaningful and ethical participation.

By refusing to conduct research when we are not the appropriate person to conduct it ethically, responsibly and competently, we may avoid contributing to the marginalisation of already marginalised communities. However, Hagen et al. (2023, p. 127) argue that researchers too often proceed despite their limitations and then try to justify their choice. They note researchers’ privileges foster a sense of entitlement to conduct research, often justified by claims that it aims to “give voice to marginalised groups,” “provide a multiplicity of perspectives,” or “do research no-one else wants to do.” However, such rhetorical devices, the authors observe, often mask more prosaic motivations; personal curiosity, the pull of academic trends or the desire to spend time in an “exotic” location. Beyond these motivations, there may also be economic imperatives such as earning an income, securing funding and maintaining employment within increasingly competitive and precarious academic labour markets. There may also be career-related pressures including “publish or perish” expectations, performance metrics and the pursuit of tenure/promotion.

### Ethical Reflexivity That Requires Knowing the Ethics of the Researched Community

Ethical reflexivity requires knowing what ethics mean to research participants and their community as ethics is shaped by community norms and cultural values (Kouritzin & Nakagawa, 2018). This is what Oyinloye calls “participant-centred ethics” (Oyinloye, 2021, 2024) or the “ethics of the researched community” (Kouritzin & Nakagawa, 2018, p. 677). It would be naïve to assume that ethical issues that arise during the research process can be handled properly without first understanding what the community considers right and wrong. How can researchers recognise and respond appropriately to ethical challenges if they do not know what is valued, respected or seen as beneficial or harmful in that community? In order to fully understand the “ethics of the researched community,” (Kouritzin & Nakagawa, 2018, p. 677) researchers need to listen to and engage with research participants, communities, local researchers and academics ideally before beginning their studies or during the design stage of their studies as is common practice in participatory research (Abebe & Refstie, 2024; Cornish et al., 2023; Vaughn & Jacquez, 2020). A university research ethics committee's protocol may not always align with a community's ethical principles. During ethnographic research with two rural Yorùbá communities in Northcentral Nigeria, Oyinloye (2021), for example, observed that British institutional and disciplinary ethics frameworks were sometimes insufficient or ill-suited for addressing situational ethical challenges in the field. In response, Oyinloye (2021) developed a “participant-centred virtue ethics approach,” known as the Ọmọlúàbí moral-ethical framework. Grounded in virtue ethics, this framework operationalises reflexivity through principles such as maintaining continuity, adhering to local and national procedures, adapting to local customs and practice and providing tangible benefits.

## Limits of Reflexivity in Community Centred Ethical Research

Reflexivity is important but, as Brown and Strega (2015, p. 6) argue, “it cannot on its own ensure the protection of marginalised communities” or ensure research outcomes that genuinely benefit them. In reviewing the literature on critiques of reflexive positionality, Gani and Khan (2024) point out several limitations of reflexivity that undermine its ability to protect research participants and communities on its own. For example, they note that, while reflexivity can help researchers recognise unfair or unequal relationships between the researcher and the researched, it cannot get rid of them. They also critique “performative declarations of positionality” (p.2), arguing that reflexivity can foreground researchers’ unease while turning participants and communities into “an instrument in the process of self-knowing” (p.6). Further, they mentioned the possibility of reflexivity becoming overly self-focused with researchers paying more attention to themselves than the research subject.

In line with this argument, Boer Cueva et al. (2024, p. 18) emphasise that recognising and reflecting on power hierarchies is a necessary foundation for ethical research. However, they argue that “the process of knowledge cultivation must include not only a commitment to reflection but also a commitment to acting on such reflection to hold ourselves and our communities accountable.” That is to say, awareness of power will not result in ethical transformation unless we do something with that awareness. Similarly, Smith (2013) notes that self-reflection alone often fails to dismantle the very structures that uphold privilege.

Extending this concern from epistemic distortion to methodological practice, Millora et al. (2020) describe reflexivity as vulnerable to becoming a “tick-box” exercise where it is done mainly to meet a formal requirement rather than to support real ethical reflection or build ongoing, meaningful relationships between researchers and participants. Dean (2021, p. 184) therefore argues that, particularly in research on social inequalities, reflexivity should be “a means to a research end, rather than a research end in itself.”

## Conclusion

Reflexivity can be useful in advancing ethical practices in studies involving marginalised groups and communities. In this paper, I have offered my reflections on what ethical reflexivity that centres the researched community might look like. However, we should not treat reflexivity as the end point of ethical research. Instead, we should see reflexivity as part of a broader commitment to accountability, non-extractive research and research that benefits participants and communities.
